# Hierarchical Wilson–Cowan Models and Connection Matrices

**DOI:** 10.3390/e25060949

**Published:** 2023-06-16

**Authors:** W. A. Zúñiga-Galindo, B. A. Zambrano-Luna

**Affiliations:** School of Mathematical & Statistical Sciences, University of Texas Rio Grande Valley, One West University Blvd., Brownsville, TX 78520, USA; wilson.zunigagalindo@utrgv.edu

**Keywords:** Wilson–Cowan model, connection matrices, p-adic numbers, small-world networks

## Abstract

This work aims to study the interplay between the Wilson–Cowan model and connection matrices. These matrices describe cortical neural wiring, while Wilson–Cowan equations provide a dynamical description of neural interaction. We formulate Wilson–Cowan equations on locally compact Abelian groups. We show that the Cauchy problem is well posed. We then select a type of group that allows us to incorporate the experimental information provided by the connection matrices. We argue that the classical Wilson–Cowan model is incompatible with the small-world property. A necessary condition to have this property is that the Wilson–Cowan equations be formulated on a compact group. We propose a *p*-adic version of the Wilson–Cowan model, a hierarchical version in which the neurons are organized into an infinite rooted tree. We present several numerical simulations showing that the *p*-adic version matches the predictions of the classical version in relevant experiments. The *p*-adic version allows the incorporation of the connection matrices into the Wilson–Cowan model. We present several numerical simulations using a neural network model that incorporates a *p*-adic approximation of the connection matrix of the cat cortex.

## 1. Introduction

This work explores the interplay among Wilson–Cowan models, connection matrices, and non-Archimedean models of complex systems.

The Wilson–Cowan model describes the evolution of excitatory and inhibitory activity in a synaptically coupled neuronal network. The model is given by the following system of non-linear integro-differential evolution equations:

$$\begin{array}{cc} \tau \frac{\partial E(x,t)}{\partial t}= & -E(x,t)+\\ \; & \\ \; & \left(1-r_{E}E\left(x,t\right)\right)S_{E}\left(w_{EE}\left(x\right)*E\left(x,t\right)-w_{EI}\left(x\right)*I\left(x,t\right)+h_{E}\left(x,t\right)\right)\\ \; & \\ \tau \frac{\partial I(x,t)}{\partial t}= & -I(x,t)+\\ \; & \\ \; & \left(1-r_{I}I\left(x,t\right)\right)S_{I}\left(w_{IE}\left(x\right)*E\left(x,t\right)-w_{II}\left(x\right)*I\left(x,t\right)+h_{I}\left(x,t\right)\right), \end{array}$$

where E(x,t) is a temporal coarse-grained variable describing the proportion of excitatory neuron firing per unit of time at position x∈R at instant $t\in R_{+}$. Similarly, the variable I(x,t) represents the activity of the inhibitory population of neurons. The main parameters of the model are the strength of the connections among the subtypes of population ($w_{EE}$, $w_{IE}$, $w_{EI}$, and $w_{II}$) and the strength of the input to each subpopulation ($h_{E}\left(x,t\right)$ and $h_{I}\left(x,t\right)$). This model generates a diversity of dynamical behaviors that are representative of activity observed in the brain, such as multistability, oscillations, traveling waves, and spatial patterns; see, e.g., [1,2,3] and the references therein.

We formulate the Wilson–Cowan model on locally compact Abelian topological groups. The classical model corresponds to the group (R,+). In this framework, using classical techniques on semilinear evolution equations (see, e.g., [4,5]), we show that the corresponding Cauchy problem is locally well posed, and if $r_{E}=r_{I}=0$, it is globally well posed; see Theorem 1. This last condition corresponds to the case of two coupled perceptrons.

Nowadays, there is a large number of experimental data about the connection matrices of the cerebral cortex of invertebrates and mammalians. Based on these data, several researchers hypothesized that cortical neural networks are arranged in fractal or self-similar patterns and have the small-world property; see, e.g., [6,7,8,9,10,11,12,13,14,15,16,17,18,19] and the references therein. Connection matrices provide a static view of neural connections.

The investigation of the relationships between the Wilson–Cowan model and connection matrices is quite natural, since the model was proposed to explain the cortical dynamics, while the matrices describe the functional geometry of the cortex. We initiate this study here.

A network having the small-world property necessarily has long-range interactions; see Section 3. In the Wilson–Cowan model, the kernels ($w_{EE}$, $w_{IE}$, $w_{EI}$, and $w_{II}$) describing the neural interactions are Gaussian in nature, so only short-range interactions may occur. For practical purposes, these kernels have compact support. On the other hand, the Wilson–Cowan model on a general group requires that the kernels be integrable; see Section 2. We argue that *G* must be compact to satisfy the small-world property. Under this condition, any continuous kernel is integrable. Wilson and Cowan formulated their model on the group (R,+). The only compact subgroup of this group is the trivial one. The small-world property is, therefore, incompatible with the classical Wilson–Cowan model.

It is worth noting that the absence of non-trivial compact subgroups in (R,+) is a consequence of the Archimedean axiom (the absolute value is not bounded on the integers). Therefore, to avoid this problem, we can replace R with a non-Archimedean field, which is a field where the Archimedean axiom is not valid. We selected the field of the *p*-adic numbers. This field has infinitely many compact subgroups, and the balls have center in the origin. We selected the unit ball, the ring of *p*-adic numbers $Z_{p}$. The *p*-adic integers are organized in an infinite rooted tree. We used this hierarchical structure as the topology for our *p*-adic version of the Wilson–Cowan model. In principle, we could use other groups, such as the classical compact groups, to replace (R,+), but it is also essential to have a rigorous study of the discretization of the model. For the group $Z_{p}$, this task can be performed using standard approximation techniques for evolutionary equations; see, e.g., [5] (Section 5.4).

The *p*-adic Wilson–Cowan model admits good discretizations. Each discretization corresponds to a system of non-linear integro-differential equations on a finite rooted tree. We show that the solution of the Cauchy problem of this discrete system provides a good approximation to the solution of the Cauchy problem of the *p*-adic Wilson–Cowan model; see Theorem 2.

We provide extensive numerical simulations of *p*-adic Wilson–Cowan models. In Section 5, we present three numerical simulations showing that the *p*-adic models provide a similar explanation to the numerical experiments presented in [2]. In these experiments, the kernels ($w_{EE}$, $w_{IE}$, $w_{EI}$, and $w_{II}$) were chosen to have properties similar to those of the kernels used in [2]. In Section 6, we consider the problem of how to integrate the connection matrices into the *p*-adic Wilson–Cowan model. This fundamental scientific task aims to use the vast number of data on maps of neural connections to understand the dynamics of the cerebral cortex of invertebrates and mammalians. We show that the connection matrix of the cat cortex can be well approximated with a *p*-adic kernel $K_{r}\left(x,y\right)$. We then replace the excitatory–excitatory relation term $w_{EE}*E$ with $\int_{Z_{p}}K_{r}\left(x,y\right)E\left(y\right)dy$ but keep the other kernels as in Simulation 1 presented in Section 5. The response of this network is entirely different from that given in Simulation 1. For the same stimulus, the response of the last network exhibits very complex patterns, while the response of the network presented in Simulation 1 is simpler.

The *p*-adic analysis has shown to be the right tool in the construction of a wide variety of models of complex hierarchic systems; see, e.g., [20,21,22,23,24,25,26,27,28] and the references therein. Many of these models involve abstract evolution equations of the type $\partial_{t}u+Au=F\left(u\right)$. In these models, the discretization of operator A is an ultrametric matrix $A_{l}={\left[a_{ij}\right]}_{i,j\in G_{l}}$, where $G_{l}$ is a finite rooted tree with *l* levels and $p^{l}$ branches; here, *p* is a fixed prime number (see the numerical simulations in [27,28]). Locally, connection matrices look very similar to matrices $A_{l}$. The problem of approximating large connection matrices with ultrametric matrices is an open problem.

## 2. An Abstract Version of the Wilson–Cowan Equations

In this section, we formulate the Wilson–Cowan model on locally compact topological groups and study the well-posedness of the Cauchy problem attached to these equations.

### 2.1. Wilson–Cowan Equations on Locally Compact Abelian Topological Groups

Let $\left(G,+\right)$ be a locally compact Abelian topological group. Let dμ be a fixed Haar measure on $\left(G,+\right)$. The basic example is $\left(R^{N},+\right)$, the *N*-dimensional Euclidean space considered an additive group. In this case, dμ is the Lebesgue measure of $R^{N}$.

Let $L^{\infty }\left(G\right)$ be the R-vector space of functions f:G→R satisfying

$${∥f∥}_{\infty }=\underset{x\in G\setminus A}{\sup}\left|f\left(x\right)\right|<\infty ,$$

where A is a subset of G with measure zero. Let $L^{1}\left(G\right)$ be the R-vector space of functions f:G→R satisfying

$${∥f∥}_{1}=\int_{G}\left|f\left(x\right)\right|d\mu <\infty .$$

For a fixed $w\in L^{1}\left(G\right)$, the mapping

$$\begin{array}{ccc} L^{\infty }\left(G\right) & \to & L^{\infty }\left(G\right)\\ f\left(x\right) & \to & \left(w*f\right)\left(x\right)=\int_{G}\;w\left(x-y\right)f\left(y\right)d\mu \left(y\right) \end{array}$$

is a well-defined, linearly bounded operator satisfying

$${∥w*f∥}_{\infty }\leq {∥w∥}_{1}{∥f∥}_{\infty }.$$

**Remark** **1.** *(i) We recall that f:R→R is called a Lipschitz function if there is a positive constant L(f) such that $\left|f(x)-f(y)\right|\leq L\left(f\right)\left|x-y\right|$ for all x and y.*

*(ii) Given X and Y, Banach spaces, we denote by C(X,Y) the space of continuous functions from X to Y.*

*(iii) If Y=R, we use the simplified notation C(X).*

We fix two bounded Lipschitz functions $S_{E}$ and $S_{I}$ satisfying

$$S_{E}\left(0\right)=S_{I}\left(0\right)=0.$$

We also fix $w_{EE}$, $w_{IE}$, $w_{EI}$, $w_{II}\in L^{1}\left(G\right)$, and $h_{E}\left(x,t\right)$, $h_{I}\left(x,t\right)\in C\left(\left[0,\infty \right],L^{\infty }\left(G\right)\right)$.

The Wilson–Cowan model on G is given by the following system of non-linear integro-differential evolution equations:

$$\begin{array}{cc} \tau \frac{\partial E(x,t)}{\partial t} & =-E(x,t)+\\ \; & \left(1-r_{E}E\left(x,t\right)\right)S_{E}\left(w_{EE}\left(x\right)*E\left(x,t\right)-w_{EI}\left(x\right)*I\left(x,t\right)+h_{E}\left(x,t\right)\right)\\ \tau \frac{\partial I(x,t)}{\partial t} & =-I(x,t)+\\ \; & \left(1-r_{I}I\left(x,t\right)\right)S_{I}\left(w_{IE}\left(x\right)*E\left(x,t\right)-w_{II}\left(x\right)*I\left(x,t\right)+h_{I}\left(x,t\right)\right), \end{array}$$

where ∗ denotes the convolution in the space variables, and $r_{E}$, $r_{I}\in R$.

The space $X:=L^{\infty }\left(G\right)\times L^{\infty }\left(G\right)$ endowed with the norm

$$∥\left(f_{1},f_{2}\right)∥=\max\left\{{∥f_{1}∥}_{\infty },{∥f_{2}∥}_{\infty }\right\}$$

is a real Banach space.

Given $f=\left(f_{1},f_{2}\right)\in X$, and P(x), $Q\left(x\right)\in L^{\infty }\left(G\right)$, we set

(1) $$F_{E}\left(f\right)=S_{E}\left(w_{EE}\left(x\right)*f_{1}\left(x\right)-w_{EI}\left(x\right)f_{2}\left(x\right)+P\left(x\right)\right),$$

and

(2) $$F_{I}\left(f\right)=S_{I}\left(w_{IE}\left(x\right)*f_{1}\left(x\right)-w_{II}\left(x\right)*f_{2}\left(x\right)+Q\left(x\right)\right).$$

We also set

$$\begin{array}{ccc} X & \to & X\\ f & \to & H(f), \end{array}$$

where $H\left(f\right)=\left(H_{E}\left(f\right),H_{I}\left(f\right)\right)$ and

(3) $$H_{E}\left(f\right)=\left(1-r_{E}f_{1}\right)F_{E}\left(f\right),\;H_{I}\left(f\right)=\left(1-r_{I}f_{2}\right)F_{I}\left(f\right).$$

**Remark** **2.** *We say that H is Lipschitz continuous (or globally Lipschitz) if there is a constant L(H) such that $∥H(f)-H(g)∥\leq L\left(H\right)∥f-g∥$, for all f, g∈X. We also say that H is locally Lipschitz continuous (or locally Lipschitz) if for every h∈X, there exists a ball $B_{R}\left(h\right)=\left\{f\in X;∥f-h∥<R\right\}$ such that $∥H(f)-H(g)∥\leq L\left(R,h\right)∥f-g∥$ for all f, $g\in B_{R}\left(h\right)$. Since X is a vector space, without loss of generality, we can assume that h=0.*

**Lemma** **1.** *We use the above notation. If $r_{I}\neq 0$ or $r_{E}\neq 0$, H:X→X is a well-defined locally Lipschitz mapping. If $r_{I}=$$r_{E}=0$, then H:X→X is a well-defined, globally Lipschitz mapping.*

**Proof.**  We first notice that for *f*, g∈X, using that $S_{E}$ is Lipschitz,

$$\begin{array}{cc} \; & \left|\left(F_{E}\left(f\right)-F_{E}\left(g\right)\right)\left(x\right)\right|\\ \; & \leq L\left(S_{E}\right)\left|w_{EE}\left(x\right)*\left(f_{1}\left(x\right)-g_{1}\left(x\right)\right)-w_{EI}\left(x\right)\left(f_{2}\left(x\right)-g_{2}\left(x\right)\right)\right|\\ \; & \leq L\left(S_{E}\right)\left\{{∥w_{EE}∥}_{1}{∥f_{1}-g_{1}∥}_{\infty }+{∥w_{EI}∥}_{1}{∥f_{2}-g_{2}∥}_{\infty }\right\}\\ \; & \leq L\left(S_{E}\right)\max\left\{{∥w_{EE}∥}_{1},{∥w_{EI}∥}_{1}\right\}∥f-g∥, \end{array}$$

which implies that

(4) $$∥F_{E}\left(f\right)-F_{E}\left(g\right)∥\leq L\left(F_{E}\right)∥f-g∥.$$

Similarly,

(5) $$∥F_{I}\left(f\right)-F_{I}\left(g\right)∥\leq L\left(F_{I}\right)∥f-g∥,$$

where $L\left(F_{I}\right)=L\left(S_{I}\right)\max\left\{{∥w_{IE}∥}_{1},{∥w_{II}∥}_{1}\right\}$.

Now, using estimation (4) and the fact that $∥F_{E}\left(f\right)∥\leq {∥S_{E}∥}_{\infty }$,

$$\begin{array}{c} ∥H_{E}\left(f\right)-H_{E}\left(g\right)∥=∥\left(1-r_{E}f_{1}\right)F_{E}\left(f\right)-\left(1-r_{E}g_{1}\right)F_{E}\left(g\right)∥=\\ ∥\left(1-r_{E}f_{1}\right)\left(F_{E}\left(f\right)-F_{E}\left(g\right)\right)-r_{E}F_{E}\left(g\right)\left(f_{1}-g_{1}\right)∥\\ \leq \left(1+\left|r_{E}\right|{∥f_{1}∥}_{\infty }\right)∥F_{E}\left(f\right)-F_{E}\left(g\right)∥+\left|r_{E}\right|∥F_{E}\left(f\right)∥{∥f_{1}-g_{1}∥}_{\infty }\\ \leq \left\{\left(1+\left|r_{E}\right|{∥f_{1}∥}_{\infty }\right)L\left(F_{E}\right)+\left|r_{E}\right|{∥S_{E}∥}_{\infty }\right\}∥f-g∥. \end{array}$$

With a similar reasoning, using estimation (5), one obtains

$${∥H_{I}\left(f\right)-H_{I}\left(g\right)∥}_{\infty }\leq \left(\left(1+\left|r_{I}\right|{∥f_{2}∥}_{\infty }\right)L\left(F_{I}\right)+\left|r_{I}\right|{∥S_{I}∥}_{\infty }\right)∥f-g∥,$$

and consequently,

(6) $$\begin{array}{cc} ∥H(f)-H(g)∥ & =\max\left\{{∥H_{E}\left(f\right)-H_{E}\left(g\right)∥}_{\infty },{∥H_{I}\left(f\right)-H_{I}\left(g\right)∥}_{\infty }\right\}\\ \; & \leq \left(A\left(1+B{∥f_{}∥}_{\infty }\right)+C\right)∥f-g∥, \end{array}$$

where

$$A:=\max\left\{L\left(F_{E}\right),L\left(F_{I}\right)\right\},B:=\left\{\left|r_{E}\right|,\left|r_{I}\right|\right\},C:=\max\left\{\left|r_{E}\right|{∥S_{E}∥}_{\infty },\left|r_{I}\right|{∥S_{I}∥}_{\infty }\right\}.$$

In the case $r_{E}=r_{I}=0$, estimation (6) takes the form

(7) $$∥H(f)-H(g)∥\leq A∥f-g∥.$$

This, in turn, implies that for f∈X,

(8) $$\begin{array}{c} ∥H(f)∥\leq ∥H(f)-H(0)∥+∥H(0)∥\leq A∥f∥+∥\left(F_{E}\left(0\right),F_{I}\left(0\right)\right)∥ \end{array}\;=A∥f∥+∥\left(S_{E}\left(0\right),S_{I}\left(0\right)\right)∥\leq A∥f∥+\max\left\{{∥S_{E}∥}_{\infty },{∥S_{I}∥}_{\infty }\right\}<\infty .$$

Then, estimations (7) and (8) imply that H is a well-defined, globally Lipschitz mapping.

We now consider the case $r_{I}\neq 0$ or $r_{E}\neq 0$. Let us take *f*, $g\in B_{R}\left(0\right)$ for some R>0. Then, ${∥f_{1}∥}_{\infty }<R$, and estimation (6) takes the form

$$\begin{array}{cc} ∥H(f)-H(g)∥ & \leq \left\{\left(1+\left|r_{E}\right|R\right)L\left(F_{E}\right)+\left|r_{E}\right|{∥S_{E}∥}_{\infty }\right\}∥f-g∥\\ \; & \leq C∥f-g∥,\;\mathrm{for}\;f,g\in B_{R}\left(0\right). \end{array}$$

This implies that

(9) $$∥H(f)∥\leq ∥H(f)-H(0)∥+∥H(0)∥\leq C∥f∥+\max\left\{{∥S_{E}∥}_{\infty },{∥S_{I}∥}_{\infty }\right\}<\infty .$$

Then, the restriction of H to $B_{R}\left(0\right)\times B_{R}\left(0\right)$ is a well-defined Lipschitz mapping. □

The estimations given in Lemma 1 are still valid for functions depending continuously on a parameter *t*. More precisely, let us take T>0 and $f_{i}\in C\left(\left[0,T\right],U\right)$, for i=1,2, where U⊂$L^{\infty }\left(G\right)$ is an open subset. We assume that

$$\left(0,T\right)\subset f_{i}^{-1}\left(U\right),\;\mathrm{for}\;i=1,2.$$

We use the notation $f_{i}=f_{i}\left(\cdot ,t\right)$, where $t\in \left[0,T\right]$ and i=1,2. We replace P(x) with $h_{E}\left(x,t\right)$ and Q(x) with $h_{I}\left(x,t\right)$, where $h_{E}\left(x,t\right)$, $h_{I}\left(x,t\right)\in C\left(\left[0,\infty \right),L^{\infty }\left(G\right)\right)$. We denote the corresponding mapping $H\left(f\right)$ by $H\left(f,s\right)$. We also set $X_{U,T}:=\left[0,T\right]\times U$.

**Lemma** **2.** *With the above notation, the following assertions hold:*

*(i)*  *The mapping $H:X_{U,T}\times X_{U,T}\to X$ is continuous, and for each $t\in \left(0,T\right)$ and each h∈U, there exist R>0 and L<∞ such that*
$$∥H\left(f,s\right)-H\left(g,s\right)∥\leq L∥f-g∥\;\mathrm{for}\;f,g\in B_{R}\left(h\right),\;s\in \left[0,t\right].$$
*(ii)*  *For $t\in \left(0,T\right)$ and f∈U×U,*
$$\int_{0}^{t}∥H\left(f,s\right)∥ds<\infty .$$

**Proof.**  (i) This follows from Lemma 1. By estimations (8) and (9), $∥H\left(f,s\right)∥$ is bounded by a positive constant *C* depending on *R*; then,

$$\int_{0}^{t}∥H\left(f,s\right)∥ds<CT.$$

 □

### 2.2. The Cauchy Problem

With the above notation, the Cauchy problem for the abstract Wilson–Cowan system takes the following form:

(10) $$\left\{\begin{array}{cc} \tau \frac{\partial }{\partial t}\left[\begin{array}{c} E\left(x,t\right)\\ I\left(x,t\right) \end{array}\right]+\left[\begin{array}{c} E\left(x,t\right)\\ I\left(x,t\right) \end{array}\right]=H\left(\left[\begin{array}{c} E\left(x,t\right)\\ I\left(x,t\right) \end{array}\right]\right), & x\in G,\;t\geq 0\\ \; & \\ \left[\begin{array}{c} E\left(x,0\right)\\ I\left(x,0\right) \end{array}\right]=\left[\begin{array}{c} E_{0}\left(x\right)\\ I_{0}\left(x\right) \end{array}\right]\in X. \end{array}\right.$$

**Theorem** **1.** *(i) There exists $T_{0}\in \left(0,T\right]$ depending on $\left[\begin{array}{c} E_{0}\left(x\right)\\ I_{0}\left(x\right) \end{array}\right]\in X$, such that Cauchy problem (10) has a unique solution $\left[\begin{array}{c} E\left(x,t\right)\\ I\left(x,t\right) \end{array}\right]$ in $C^{1}\left(\left[0,T_{0}\right),X\right)$.*

*(ii) The solution satisfies*

(11) $$\begin{array}{c} E\left(x,t\right)=e^{\frac{-t}{\tau }}E_{0}\left(x\right)+\int_{0}^{t}e^{\frac{-(t-s)}{\tau }}\left(1-r_{E}E\left(x,s\right)\right)\times \\ \left\{S_{E}\left(w_{EE}\left(x\right)*E\left(x,s\right)-w_{EI}\left(x\right)*I\left(x,s\right)+h_{E}\left(x,s\right)\right)\right\}ds, \end{array}$$

(12) $$\begin{array}{c} I\left(x,t\right)=e^{\frac{-t}{\tau }}I_{0}\left(x\right)+\int_{0}^{t}e^{\frac{-(t-s)}{\tau }}\left(1-r_{E}I\left(x,s\right)\right)\times \\ \left\{S_{I}\left(w_{IE}\left(x\right)*E\left(x,s\right)-w_{II}\left(x\right)*I\left(x,s\right)+h_{E}\left(x,s\right)\right)\right\}ds, \end{array}$$

*for $t\in \left[0,T_{0}\right)$ and x∈G.*

*(iii) If $r_{I}=$$r_{E}=0$, then $T_{0}=\infty$ for any $\left[\begin{array}{c} E_{0}\left(x\right)\\ I_{0}\left(x\right) \end{array}\right]\in X$, and*

(13) $$\left|E\left(x,t\right)\right|\leq {∥E_{0}∥}_{\infty }+\tau {∥S_{E}∥}_{\infty }\;\;\mathrm{and}\;\;\left|I\left(x,t\right)\right|\leq {∥I_{0}∥}_{\infty }+\tau {∥S_{I}∥}_{\infty }.$$

*(iv) The solution $\left[\begin{array}{c} E\left(x,t\right)\\ I\left(x,t\right) \end{array}\right]$ in $C^{1}\left(\left[0,T_{0}\right),X\right)$ depends continuously on the initial value.*

**Proof.**  (i)–(iii) By Lemma 2-(i) and [5] (Lemma 5.2.1 and Theorem 5.1.2), for each

$$\left[\begin{array}{c} E_{0}\left(x\right)\\ I_{0}\left(x\right) \end{array}\right]\in X,$$

there exists a unique $\left[\begin{array}{c} E\left(x,t\right)\\ I\left(x,t\right) \end{array}\right]\in C\left(\left[0,T_{0}\right],X\right)$ that satisfies (11) and (12). By Lemma 2-(i) and [5] (Corollary 4.7.5), $\left[\begin{array}{c} E\left(x,t\right)\\ I\left(x,t\right) \end{array}\right]\in C^{1}\left(\left[0,T_{0}\right),X\right)$ and satisfies (10). By [4] (Theorem 4.3.4) (see also [5] (Theorem 5.2.6)), $T_{0}=\infty$ or $T_{0}<\infty$ and $\lim_{t\to T_{0}}∥\left(E\left(t\right),I\left(t\right)\right)∥=\infty$. In the case $r_{I}=$$r_{E}=0$, by using

$$\begin{array}{c} \int_{0}^{t}e^{\frac{-(t-s)}{\tau }}\left|S_{E}\left(w_{EE}\left(x\right)*E\left(x,s\right)-w_{EI}\left(x\right)*I\left(x,s\right)+h_{E}\left(x,s\right)\right)\right|ds\\ \;\;\;\;\;\;\;\;\;\;\;\;\;\;\;\;\;\;\;\;\;\;\;\;\;\;\leq {∥S_{E}∥}_{\infty }\int_{0}^{t}e^{\frac{-(t-s)}{\tau }}ds<\tau {∥S_{E}∥}_{\infty }, \end{array}$$

and

$$\begin{array}{c} \int_{0}^{t}e^{\frac{-(t-s)}{\tau }}\left|S_{I}\left(w_{IE}\left(x\right)*E\left(x,s\right)-w_{II}\left(x\right)*I\left(x,s\right)+h_{I}\left(x,s\right)\right)\right|ds\\ \;\;\;\;\;\;\;\;\;\;\;\;\;\;\;\;\;\;\;\;\;\;\;\;\;\;\;\;\;\;\;\;<\tau {∥S_{I}∥}_{\infty }, \end{array}$$

one shows (13), which implies that $T_{0}=\infty$.

(iv) This follows from [5] (Lemma 5.2.1 and Theorem 5.2.4). □

## 3. Small-World Property and Wilson–Cowan Models

After formulating the Wilson–Cowan model on locally compact Abelian groups, our next step is to find the groups for which the model is compatible with the description of the cortical networks given by connection matrices. From now on, we take $r_{I}=$$r_{E}=0$; in this case, the Wilson–Cowan equations describe two coupled perceptrons.

### 3.1. Compactness and Small-World Networks

The original Wilson–Cowan model is formulated on (R,+). The kernels $w_{AB}$, *A*, $B\in \left\{E,I\right\}$, which control the connections among neurons, are supposed to be radial functions of the form

(14) $$e^{-C_{AB}\left|x-y\right|},\;\mathrm{or}\;e^{-D_{AB}{\left|x-y\right|}^{2}},$$

where $C_{AB}$ and $D_{AB}$ are positive constants. Since R is unbounded, hypothesis (14) implies that only short-range interactions among neurons occur. The strength of the connections produced by kernels of type (14) is negligible outside of a compact set; then, for practical purposes, interactions among groups of neurons only occur at small distances.

Nowadays, it is widely accepted that the brain is a small-world network; see, e.g., [8,9,10,11] and the references therein. Small-worldness is believed to be a crucial aspect of efficient brain organization that confers significant advantages in signal processing; furthermore, small-world organization is deemed essential for healthy brain function (see, e.g., [10], and the references therein). A small-world network has a topology that produces short paths across the whole network, i.e., given two nodes, there is a short path between them (the “six degrees of separation” phenomenon). In turn, this implies the existence of long-range interactions in the network. The compatibility of the Wilson–Cowan model with the small-world network property requires a non-negligible interaction between any two groups of neurons, i.e., $w_{AB}\left(x\right)>\varepsilon >0$, for any x∈G, and for A, $B\in \left\{E,I\right\}$, where the constant ε>0 is independent of *x*. By Theorem 1, it is reasonable to expect that $w_{AB}$, *A*, $B\in \left\{E,I\right\}$ are integrable; then, necessarily, G must be compact.

Finally, we mention that $\left(R^{N},+\right)$ does not have non-trivial compact subgroups. Indeed, if $x_{0}\neq 0$, then $\langle x_{0}\rangle =\left\{nx_{0};n\in Z\right\}$ is a non-compact subgroup of $\left(R^{N},+\right)$, because $\left\{\left|n\right|;n\in Z\right\}$ is not bounded. This last assertion is equivalent to the Archimedean axiom of real numbers. In conclusion, the compatibility between the Wilson–Cowan model and the small-world property requires changing $\left(R,+\right)$ to a compact Abelian group. The simplest solution is to replace $\left(R,\left|\cdot \right|\right)$ with a non-Archimedean field $\left(F,{\left|\cdot \right|}_{F}\right)$, where the norm satisfies

$${\left|x+y\right|}_{F}\leq \max\left\{{\left|x\right|}_{F},{\left|y\right|}_{F}\right\}.$$

### 3.2. Neuron Geometry and Discreteness

Nowadays, there are extensive databases of neuronal wiring diagrams (connection matrices) of the invertebrates’ and mammalians’ cerebral cortex. The connection matrices are adjacency matrices of weighted directed graphs, where the vertices represent neurons, regions in a cortex, or neuron populations. These matrices correspond to the kernels $w_{AB}$, *A*, $B\in \left\{E,I\right\}$; then, it seems natural to consider using discrete Wilson–Cowan models [2,3] (Chapter 2). We argue that two difficulties appear. First, since the connection matrices may be extremely large, studying the corresponding Wilson–Cowan equations is only possible via numerical simulations. Second, it seems that the discrete Wilson–Cowan model is not a good approximation of the continuous Wilson–Cowan model; see [3] (page 57). Wilson–Cowan equations can be formally discretized by replacing integrals with finite sums. However, these discrete models are relevant only when they are good approximations of continuous models. Finally, we want to mention that O. Sporns has proposed the hypothesis that cortical connections are arranged in hierarchical self-similar patterns [8].

## 4. p-Adic Wilson–Cowan Models

The previous section shows that the classical Wilson–Cowan can be formulated on a large class of topological groups. This formulation does not use any information about the geometry of the neural interaction, which is encoded in the geometry of the group G. The next step is to incorporate the connection matrices into the Wilson–Cowan model, which requires selecting a specific group. In this section, we propose the *p*-adic Wilson–Cowan models where G is the ring of *p*-adic integers $Z_{p}$.

### 4.1. The *p*-Adic Integers

This section reviews some basic results of *p*-adic analysis required in this article. For a detailed exposition on *p*-adic analysis, the reader may consult [29,30,31,32]. For a quick review of *p*-adic analysis, the reader may consult [33].

From now on, *p* denotes a fixed prime number. The ring of *p*-adic integers $Z_{p}$ is defined as the completion of the ring of integers Z with respect to the *p*-adic norm ${\left|\cdot \right|}_{p}$, which is defined as

(15) $${\left|x\right|}_{p}=\left\{\begin{array}{cc} 0 & \mathrm{if}\;x=0\\ p^{-\gamma } & \mathrm{if}\;x=p^{\gamma }a\in Z, \end{array}\right.$$

where *a* is an integer coprime with *p*. The integer $\gamma =ord_{p}\left(x\right):=ord\left(x\right)$, with ord(0):=+∞, is called the *p*-*adic order* of *x*.

Any non-zero *p*-adic integer *x* has a unique expansion of the form

$$x=x_{k}p^{k}+x_{k+1}p^{k+1}+\ldots ,\;$$

with $x_{k}\neq 0$, where *k* is a non-negative integer, and $x_{j}$ are numbers from the set $\left\{0,1,\ldots ,p-1\right\}$. There are natural field operations, sum and multiplication, on *p*-adic integers; see, e.g., [34]. Norm (15) extends to $Z_{p}$ as ${\left|x\right|}_{p}=p^{-k}$ for a non-zero *p*-adic integer *x*.

The metric space $\left(Z_{p},{\left|\cdot \right|}_{p}\right)$ is a complete ultrametric space. Ultrametric means that ${\left|x+y\right|}_{p}\leq \max\left\{{\left|x\right|}_{p},{\left|y\right|}_{p}\right\}$. As a topological space, $Z_{p}$ is homeomorphic to a Cantor-like subset of the real line; see, e.g., [29,30,35].

For r∈N, let us denote by $B_{-r}\left(a\right)=\left\{x\in Z_{p};{\left|x-a\right|}_{p}\leq p^{-r}\right\}$ *the ball of radius* $p^{-r}$ *with center in* $a\in Z_{p}$ and take $B_{-r}\left(0\right):=B_{-r}$. Ball $B_{0}$ equals *the ring ofp*-*adic integers* $Z_{p}$. We use $\Omega \left(p^{r}{\left|x-a\right|}_{p}\right)$ to denote the characteristic function of ball $B_{-r}\left(a\right)$. Given two balls in $Z_{p}$, either they are disjoint, or one is contained in the other. The balls are compact subsets; thus, $\left(Z_{p},{\left|\cdot \right|}_{p}\right)$ is a compact topological space.

#### Tree-like Structures

**Figure 1:**
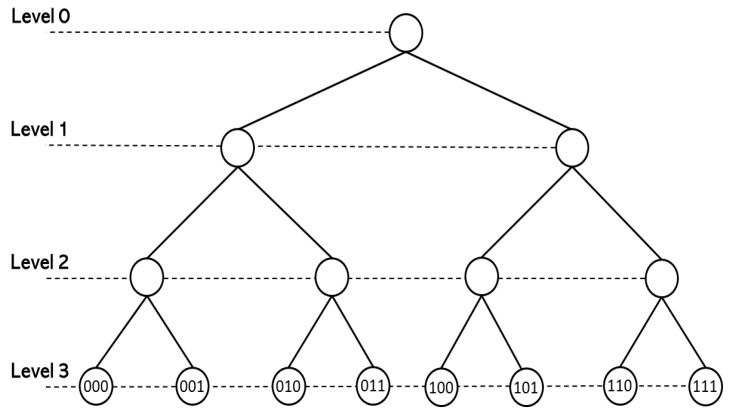
The rooted tree associated with the group $Z_{2}/2^{3}Z_{2}$. The elements of $Z_{2}/2^{3}Z_{2}$ have the form $i=i_{0}+i_{1}2+i_{2}2^{2}$,$\;i_{0}$, $i_{1}$, $i_{2}\in \left\{0,1\right\}$. The distance satisfies $-\log_{2}{\left|i-j\right|}_{2}=$ level of the first common ancestor of *i*, *j*.

The set of *p*-adic integers modulo $p^{l}$, l≥1, consists of all the integers of the form $i=i_{0}+i_{1}p+\ldots +i_{l-1}p^{l-1}$. These numbers form a complete set of representatives of the elements of additive group $G_{l}=Z_{p}/p^{l}Z_{p}$, which is isomorphic to the set of integers $Z/p^{l}Z$ (written in base *p*) modulo $p^{l}$. By restricting ${\left|\cdot \right|}_{p}$ to $G_{l}$, it becomes a normed space, and ${\left|G_{l}\right|}_{p}=\left\{0,p^{-\left(l-1\right)},\cdots ,p^{-1},1\right\}$. With the metric induced by ${\left|\cdot \right|}_{p}$, $G_{l}$ becomes a finite ultrametric space. In addition, $G_{l}$ can be identified with the set of branches (vertices at the top level) of a rooted tree with l+1 levels and $p^{l}$ branches. By definition, the tree’s root is the only vertex at level 0. There are exactly *p* vertices at level 1, which correspond with the possible values of the digit $i_{0}$ in the *p*-adic expansion of *i*. Each of these vertices is connected to the root by a non-directed edge. At level *k*, with 2≤k≤l+1, there are exactly $p^{k}$ vertices, and each vertex corresponds to a truncated expansion of *i* of the form $i_{0}+\cdots +i_{k-1}p^{k-1}$. The vertex corresponding to $i_{0}+\cdots +i_{k-1}p^{k-1}$ is connected to a vertex $i_{0}^{\prime }+\cdots +i_{k-2}^{\prime }p^{k-2}$ at level k−1 if and only if $\left(i_{0}+\cdots +i_{k-1}p^{k-1}\right)-\left(i_{0}^{\prime }+\cdots +i_{k-2}^{\prime }p^{k-2}\right)$ is divisible by $p^{k-1}$. See Figure 1. Balls $B_{-r}\left(a\right)=a+p^{r}Z_{p}$ are infinite rooted trees.

### 4.2. The Haar Measure

Since $\left(Z_{p},+\right)$ is a compact topological group, there exists a Haar measure dx, which is invariant under translations, i.e., d(x+a)=dx [36]. If we normalize this measure by the condition $\int_{Z_{p}}dx=1$, then dx is unique. It follows immediately that

$$\int_{B_{-r}\left(a\right)}dx=\int_{a+p^{r}Z_{p}}dx=p^{-r}\int_{Z_{p}}dy=p^{-r},\;r\in N.$$

In a few occasions, we use the two-dimensional Haar measure dxdy of the additive group $\left(Z_{p}\times Z_{p},+\right)$ to normalize this measure by the condition $\int_{Z_{p}}\int_{Z_{p}}dxdy=1$. For a quick review of the integration in the *p*-adic framework, the reader may consult [33] and the references therein.

### 4.3. The Bruhat–Schwartz Space in the Unit Ball

A real-valued function φ defined on $Z_{p}$ is *called Bruhat–Schwartz function (or a test function)* if, for any $x\in Z_{p}$, there exists an integer l∈N such that

(16) $$\varphi \left(x+x^{\prime }\right)=\varphi \left(x\right)\;\mathrm{for}\;\mathrm{any}\;x^{\prime }\in B_{l}.$$

The R-vector space of Bruhat–Schwartz functions supported in the unit ball is denoted by $D(Z_{p})$. For $\varphi \in D(Z_{p})$, the largest number l=l(φ) satisfying (16) is called *the exponent of local constancy (or the parameter of constancy) of* φ. A function φ in $D(Z_{p})$ can be written as

$$\varphi \left(x\right)=\sum_{j=1}^{M}\varphi \left({\tilde{x}}_{j}\right)\Omega \left(p^{-r_{j}}{\left|x-{\tilde{x}}_{j}\right|}_{p}\right),$$

where ${\tilde{x}}_{j}$, j=1,…,M, are points in $Z_{p}$; $r_{j}$, j=1,…,M, are non-negative integers; and $\Omega \left(p^{r_{j}}{\left|x-{\tilde{x}}_{j}\right|}_{p}\right)$ denotes the characteristic function of ball $B_{-r_{j}}\left({\tilde{x}}_{j}\right)={\tilde{x}}_{j}+p^{r_{j}}Z_{p}$.

We denote by $D^{l}\left(Z_{p}\right)$ the R-vector space of all test functions of the form

$$\varphi \left(x\right)=\sum_{i\in G_{l}}\varphi \left(i\right)\Omega \left(p^{l}{\left|x-i\right|}_{p}\right),\;\;\varphi \left(i\right)\in R,$$

where $i=i_{0}+i_{1}p+\ldots +i_{l-1}p^{l-1}\in G_{l}=Z_{p}/p^{l}Z_{p}$, l≥1. Notice that φ is supported on $Z_{p}$ and that $D\left(Z_{p}\right)=\cup_{l\in N}D^{l}\left(Z_{p}\right)$.

The space $D^{l}\left(Z_{p}\right)$ is a finite-dimensional vector space spanned by the basis

$${\left\{\Omega \left(p^{l}{\left|x-i\right|}_{p}\right)\right\}}_{i\in G_{l}}.$$

By identifying $\varphi \in D^{l}\left(Z_{p}\right)$ with the column vector ${\left[\varphi \left(i\right)\right]}_{i\in G_{l}}\in R^{\#G_{l}}$, we get that $D^{l}\left(Z_{p}\right)$ is isomorphic to $R^{\#G_{l}}$ endowed with the norm

$$∥{\left[\varphi \left(i\right)\right]}_{i\in G_{l}^{N}}∥=\max_{i\in G_{l}}\left|\varphi \left(i\right)\right|.$$

Furthermore,

$$D^{l}↪D^{l+1}↪D\left(Z_{p}\right),$$

where ↪ denotes continuous embedding.

### 4.4. The *p*-Adic Version and Discrete Version of the Wilson–Cowan Models

The *p*-adic Wilson–Cowan model is obtained by taking $G=Z_{p}$ and dμ=dx in (10).

On the other hand, ${∥f∥}_{1}\leq {∥f∥}_{\infty }$, and

$$L^{1}\left(Z_{p}\right)⊇L^{\infty }\left(Z_{p}\right)⊇C\left(Z_{p}\right)⊇D\left(Z_{p}\right),$$

where $C(Z_{p})$ denotes the R-space of continuous functions on $Z_{p}$ endowed with the norm ${∥\cdot ∥}_{\infty }$. Furthermore, $D(Z_{p})$ is dense in $L^{1}\left(Z_{p}\right)$ [30] (Proposition 4.3.3); consequently, it is also dense in $L^{\infty }\left(Z_{p}\right)$ and $C(Z_{p})$.

For the sake of simplicity, we assume that $w_{EE}$, $w_{IE}$, $w_{EI}$, $w_{II}\in C\left(Z_{p}\right)$, and $h_{E}\left(x,t\right)$, $h_{I}\left(x,t\right)\in C\left(\left[0,\infty \right],C\left(Z_{p}\right)\right)$. Theorem 1 is still valid under these hypotheses. We use the theory of approximation of evolution equations to construct good discretizations of the *p*-adic Wilson–Cowan system; see, e.g., [5] (Section 5.4).

This theory requires the following hypotheses.

(**A**) (a) $X=\left(C\left(Z_{p}\right)\times C\left(Z_{p}\right)\right)$ and $X_{l}=\left(D^{l}\left(Z_{p}\right)\times D^{l}\left(Z_{p}\right)\right)$, l≥1, endowed with the norm $∥f∥=∥\left(f_{1},f_{2}\right)∥=\max\left\{{∥\left(f_{1}\right)∥}_{\infty },{∥\left(f_{2}\right)∥}_{\infty }\right\}$ are Banach spaces. It is relevant to mention that $X_{l}$ is a subspace of X and that $X_{l}$ is a subspace of $X_{l+1}$.

(b) The operator

$$\begin{array}{cccc} P_{l}: & X & \to & X_{l}\\ & f\left(x\right) & \to & \left(P_{l}f\right)\left(x\right)=\sum_{i\in G_{l}}f\left(i\right)\Omega \left(p^{l}{\left|x-i\right|}_{p}\right) \end{array}$$

is linear and bounded, i.e., $P_{l}\in B\left(X,X_{l}\right)$ and $∥P_{l}f∥\leq ∥f∥$, for every f∈X.

(c) We set $1_{l}:X_{l}\to X$ to be the identity operator. Then, $1_{l}\in B\left(X_{l},X\right)$, and $∥1_{l}f∥=∥f∥$, for every $f\in X_{l}$.

(d) $P_{l}1_{l}f=f$, for l≥1, $f\in X_{l}$.

(**B**, **C**) The Wilson–Cowan system, see (10), involves the operator $\frac{1}{\tau }1$, where 1∈B(X,X) is the identity operator. As approximation, we use $1\in B(X_{l},X_{l})$, for every l≥1. Furthermore,

$$\lim_{l\to \infty }∥P_{l}f-f∥=0,$$

(see [37] (Lemma 1)).

(**D**) For $t\in \left(0,\infty \right)$, $\frac{1}{\tau }H\left(s,f\right):\left[0,t\right]\times X\to X$ is continuous and such that, for some L<∞,

$$∥\frac{1}{\tau }H\left(s,f\right)-\frac{1}{\tau }H\left(s,g\right)∥\leq L∥f-g∥,$$

for 0≤s≤t, *f*, g∈X. This assertion is a consequence of the fact that H:X→X is well-defined, globally Lipschitz; see Lemma 1.

We use the notation $E\left(t\right)=E\left(\cdot ,t\right)$, $I\left(t\right)=I\left(\cdot ,t\right)\in C^{1}\left(\left[0,T\right),X\right)$ and, for the approximations, $E_{l}\left(t\right)=E_{l}\left(\cdot ,t\right)$, $I_{l}\left(t\right)=I_{l}\left(\cdot ,t\right)\in C^{1}\left(\left[0,T\right),X\right)$. The space discretization of *p*-adic Wilson–Cowan system (10) is

(17) $$\left\{\begin{array}{c} \frac{\partial }{\partial t}\left[\begin{array}{c} E_{l}\left(t\right)\\ I_{l}\left(t\right) \end{array}\right]+\frac{1}{\tau }\left[\begin{array}{c} E_{l}\left(t\right)\\ I_{l}\left(t\right) \end{array}\right]=\frac{1}{\tau }P_{l}\left(H\left(\left[\begin{array}{c} E_{l}\left(t\right)\\ I_{l}\left(t\right) \end{array}\right]\right)\right),\\ \left[\begin{array}{c} E_{l}\left(0\right)\\ I_{l}\left(0\right) \end{array}\right]=P_{l}\left(\left[\begin{array}{c} E_{0}\left(x\right)\\ I_{0}\left(x\right) \end{array}\right]\right)\in X_{l}. \end{array}\right.$$

The next step is to obtain an explicit expression for the space discretization given in (17). We need the following formulae.

**Remark** **3.** *Let us take*

$$w\left(x\right)=\sum_{j\in G_{l}}w\left(j\right)\Omega \left(p^{l}{\left|x-j\right|}_{p}\right),\;\phi \left(y\right)=\sum_{i\in G_{l}}\phi \left(i\right)\Omega \left(p^{l}{\left|y-i\right|}_{p}\right)\in D^{l}\left(Z_{p}\right).$$

*Then,*

$$\begin{array}{c} \left(w*\phi \right)\left(x\right)=\int_{Z_{p}}w\left(x-y\right)\phi \left(y\right)dy=\\ \;\;\;\;\;\;\;\;\;\;\;\;\;\;\;\;\;\;\sum_{k\in G_{l}}\left\{p^{-l}\sum_{i\in G_{l}}w\left(k-i\right)\phi \left(i\right)\right\}\Omega \left(p^{l}{\left|x-k\right|}_{p}\right)\in D^{l}\left(Z_{p}\right). \end{array}$$

*Indeed,*

$$\left(w*\phi \right)\left(x\right)=\sum_{j\in G_{l}}\;\sum_{i\in G_{l}}w\left(j\right)\phi \left(i\right)\int_{Z_{p}}\Omega \left(p^{l}{\left|x-y-j\right|}_{p}\right)\Omega \left(p^{l}{\left|y-i\right|}_{p}\right)dy.$$

*By changing variables as z=y−i, dz=dx, in the integral,*

$$\begin{array}{cc} \left(w*\phi \right)\left(x\right) & =\sum_{j\in G_{l}}\;\sum_{i\in G_{l}}w\left(j\right)\phi \left(i\right)\int_{Z_{p}}\Omega \left(p^{l}{\left|x-z-\left(i+j\right)\right|}_{p}\right)\Omega \left(p^{l}{\left|z\right|}_{p}\right)dz\\ \; & =\sum_{j\in G_{l}}\;\sum_{i\in G_{l}}w\left(j\right)\phi \left(i\right)\int_{p^{l}Z_{p}}\Omega \left(p^{l}{\left|x-z-\left(i+j\right)\right|}_{p}\right)dz. \end{array}$$

*Now, by taking k=i+j and using the fact that $G_{l}$ is an additive group,*

$$\begin{array}{cc} \left(w*\phi \right)\left(x\right) & =\sum_{k\in G_{l}}\;\sum_{i\in G_{l}}w\left(k-i\right)\phi \left(i\right)\int_{p^{l}Z_{p}}\Omega \left(p^{l}{\left|x-z-k\right|}_{p}\right)dz\\ \; & =\sum_{k\in G_{l}}\left\{p^{-l}\sum_{i\in G_{l}}w\left(k-i\right)\phi \left(i\right)\right\}\Omega \left(p^{l}{\left|x-k\right|}_{p}\right). \end{array}$$

**Remark** **4.** *Let us take S:R→R. Then,*

$$S\left(\sum_{i\in G_{l}}\phi \left(i\right)\Omega \left(p^{l}{\left|y-i\right|}_{p}\right)\right)=\sum_{i\in G_{l}}S\left(\phi \left(i\right)\right)\Omega \left(p^{l}{\left|y-i\right|}_{p}\right).$$

*This formula follows from the fact that the supports of the functions $\Omega \left(p^{l}{\left|y-i\right|}_{p}\right)$, $i\in G_{l}$, are disjoint.*

The space discretization of the integro-differential equation in (17) is obtained by computing the term $P_{l}\left(H(\left[\begin{array}{c} E_{l}\left(t\right)\\ I_{l}\left(t\right) \end{array}\right])\right)$ using Remarks 3 and 4. By using the notation

$$w_{l}^{AB}={\left[w_{i}^{AB}\right]}_{i\in G_{l}},\;w_{i}^{AB}=w_{AB}\left(i\right),\;\mathrm{for}\;A,B\in \left\{E,I\right\},$$

$$E_{l}\left(t\right)={\left[E_{i}\left(t\right)\right]}_{i\in G_{l}},\;E_{i}\left(t\right)=E\left(i,t\right),\;\mathrm{and}\;I_{l}\left(t\right)={\left[I_{i}\left(t\right)\right]}_{i\in G_{l}},\;I_{i}\left(t\right)=I\left(i,t\right),$$

$$h_{l}^{A}\left(t\right)={\left[h_{i}^{A}\left(t\right)\right]}_{i\in G_{l}},\;h_{i}^{A}\left(t\right)=h_{A}\left(i,t\right),\;\mathrm{for}\;A\in \left\{E,I\right\},$$

and for $\phi_{l}={\left[\phi_{i}\right]}_{i\in G_{l}}$, $\theta_{l}={\left[\theta_{i}\right]}_{i\in G_{l}}$,

$$\phi_{l}*\theta_{l}={\left[\;\sum_{k\in G_{l}}\phi_{i-k}\theta_{k}\right]}_{i\in G_{l}}.$$

With this notation, the announced discretization takes the following form:

$$\left\{\begin{array}{cc} \tau \frac{\partial E_{l}\left(t\right)}{\partial t}= & -E_{l}\left(t\right)+S_{E}\left(w_{l}^{EE}*E_{l}\left(t\right)-w_{l}^{EI}*I_{l}\left(t\right)+h_{l}^{E}\left(t\right)\right)\\ \tau \frac{\partial I_{l}\left(t\right)}{\partial t}= & -I_{l}\left(t\right)+S_{I}\left(w_{l}^{IE}*E_{l}\left(t\right)-w_{l}^{II}\left(x\right)*I_{l}\left(t\right)+h_{l}^{I}\left(t\right)\right). \end{array}\right.$$

**Theorem** **2.** *Let us take $r_{I}=$$r_{E}=0$, $\left[\begin{array}{c} E_{0}\left(x\right)\\ I_{0}\left(x\right) \end{array}\right]\in X$, and $T\in \left(0,\infty \right)$. Let $\left[\begin{array}{c} E\left(t\right)\\ I\left(t\right) \end{array}\right]\in$$C^{1}\left(\left[0,T_{0}\right),X\right)$ be solutions (11) and (12) given in Theorem 1. Let $\left[\begin{array}{c} E_{l}\left(t\right)\\ I_{l}\left(t\right) \end{array}\right]$ be the solution of Cauchy problem (17). Then,*

$$\lim_{l\to \infty }\underset{0\leq t\leq T}{\sup}∥\left[\begin{array}{c} E_{l}\left(t\right)\\ I_{l}\left(t\right) \end{array}\right]-\left[\begin{array}{c} E\left(t\right)\\ I\left(t\right) \end{array}\right]∥=0.$$

**Proof.**  We first notice that Theorem 1 is valid for Cauchy problem (17); more precisely, this problem has a unique solution $\left[\begin{array}{c} E_{l}\left(t\right)\\ I_{l}\left(t\right) \end{array}\right]$ in $C^{1}\left(\left[0,T_{0}\right),X_{l}\right)$ satisfying properties akin to the ones stated in Theorem 1. Since $X_{l}$ is a subspace of X, by applying Theorem 1 to Cauchy problem (17), we obtain the existence of a unique solution $\left[\begin{array}{c} E_{l}\left(t\right)\\ I_{l}\left(t\right) \end{array}\right]$ in $C^{1}\left(\left[0,T_{0}\right),X\right)$ satisfying the properties announced in Theorem 1. To show that the solution $\left[\begin{array}{c} E_{l}\left(t\right)\\ I_{l}\left(t\right) \end{array}\right]$ belongs to $C(\left[0,T_{0}\right),X_{l})$, we use [5] (Theorem 5.2.2). For similar reasoning, the reader may consult Remark 2 and the proof of Theorem 1 in [27]. The proof of the theorem follows from hypotheses A, B, C, and D according to [5] (Theorem 5.4.7). For similar reasoning, the reader may consult the proof of Theorem 4 in [27]. □

## 5. Numerical Simulations

We use heat maps to visualize approximations of the solutions of *p*-adic discrete Wilson–Cowan Equations (17). The vertical axis gives the position, which is a truncated *p*-adic number. These numbers correspond to a rooted tree’s vertices at the top level, i.e., $G_{l}$; see Figure 1. For convenience, we include a representation of this tree. The heat maps’ colors represent the solutions’ values in a particular neuron. For instance, let us take p=2, l=4, and

(18) $$\phi \left(x\right)=\Omega (2^{4}{\left|x\right|}_{2})-\Omega (2^{4}{\left|x-2\right|}_{2})+\Omega (2^{4}{\left|x-1\right|}_{2})+\Omega (2^{4}{\left|x-7\right|}_{2}).$$

**Figure 2:**
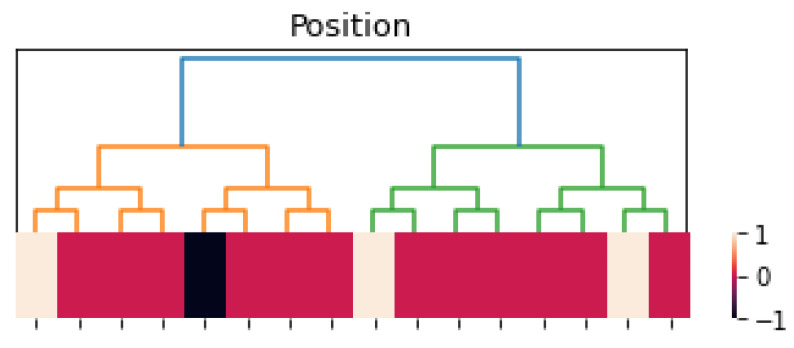
Heat map of function ϕ(x); see (18). Here, ϕ(0)=ϕ(1)=ϕ(7)=1 is white; ϕ(2)=−1 is black; and ϕ(x)=0 is red for x≠0,1,7,2.

The corresponding heat map is shown in Figure 2. If the function depends on two variables, say, ϕ(x,t), where $x\in Z_{p}$ and t∈R, the corresponding heat map color represents the value of ϕ(x,t) at time *t* and neuron *x*.

We take τ=10, $r_{I}=r_{E}=1$, p=3, and l=6; then,

$$w_{AB}\left(x\right)=b_{AB}\exp\left(\sigma_{AB}\right)-b_{AB}\exp(\sigma_{AB}{\left|x\right|}_{p}),\;\mathrm{for}\;A,B\in \left\{E,I\right\},$$

and

$$S_{A}\left(z\right)=\frac{1}{1+\exp(-v_{A}\left(z-\theta_{A}\right))}-\frac{1}{1+\exp(v_{A}\theta_{A})},\;\mathrm{for}\;z\in R,\;A\in \left\{E,I\right\}.$$

The kernel $w_{AB}\left(x\right)$ is a decreasing function of ${\left|x\right|}_{p}$. Thus, close neurons interact strongly. $S_{A}\left(z\right)$ is a sigmoid function satisfying $S_{A}\left(0\right)=0$.

### 5.1. Numerical Simulation 1

**Figure 3:**
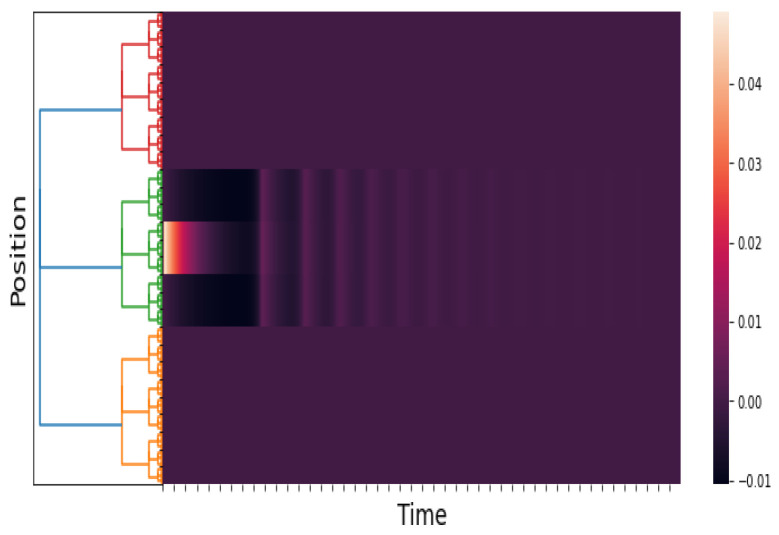
An approximation of E(x,t). We take Q=0 and δ=5. The time axis goes from 0 to 100 with a step of 0.05. The figure shows the response of the network to a brief localized stimulus (the pulse given in (19)). The response is also a pulse. This result is consistent with the numerical results in [2] (Section 2.2.1, Figure 3).

**Figure 4:**
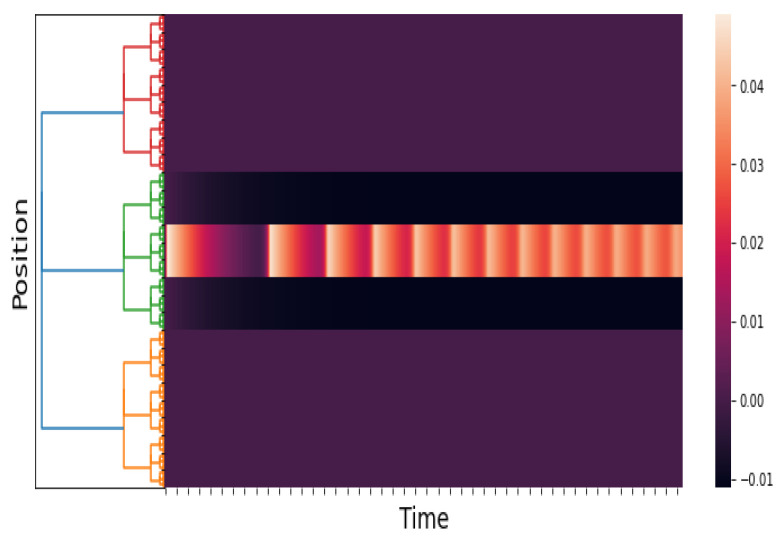
An approximation of E(x,t). We take Q=0 and δ=100. The time axis goes from 0 to 200 with a step of 0.05. The figure shows the response of the network to a maintained stimulus (see (19)). The response is a pulse train. This result is consistent with the numerical results in [2] (Section 2.2.5, Figure 7).

**Figure 5:**
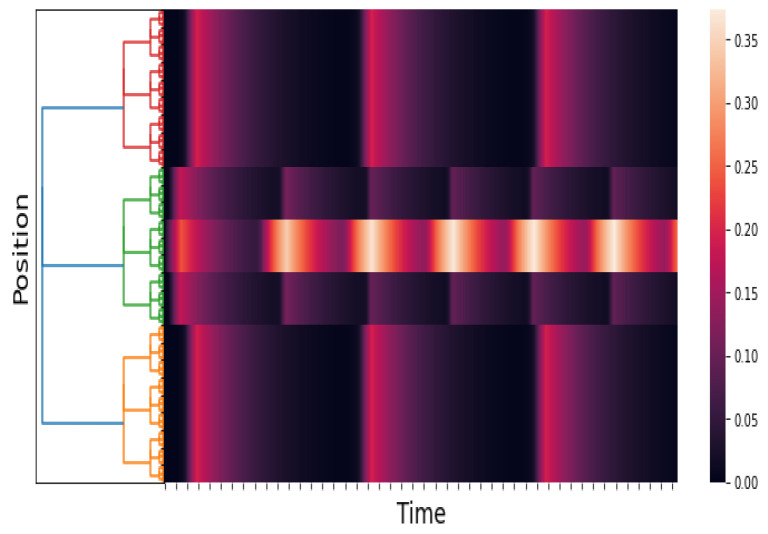
An approximation of E(x,t). We take Q=−30 and δ=100. The time axis goes from 0 to 100 with a step of 0.05. The figure shows the response of the network to a maintained stimulus (see (19) and (20)). The response is a pulse train in space and time. This result is consistent with the numerical results in [2] (Section 2.2.7, Figure 9).

The purpose of this experiment is to show the response of the *p*-adic Wilson–Cowan network to a short pulse and a constant stimulus. See Figure 3, Figure 4 and Figure 5. Our results are consistent with the results obtained by Cowan and Wilson in [2] (Sections 2.2.1–2.2.5). The pulses are

(19) $$h_{E}\left(x,t\right)=3.7\Omega (p^{2}{\left|x-4\right|}_{p})1_{\left[0,\delta \right]}\left(t\right),\;\mathrm{for}\;x\in Z_{p},\;t\in \left[0,\delta \right],$$

(20) $$h_{I}\left(x,t\right)={Q\Omega (|x-4|}_{p})1_{\left[0,\delta \right]}\left(t\right),\;\mathrm{for}\;x\in Z_{p},\;t\in \left[0,\delta \right],$$

where $1_{\left[0,\delta \right]}\left(t\right)$ is the characteristic function of time interval $\left[0,\delta \right]$, δ>0. We use the following parameters: $v_{E}=2.75$, $v_{I}=0.3$, $b_{EE}=1.5$, $\sigma_{EE}=4$, $b_{II}=1.8$, $\sigma_{II}=3$, $\theta_{E}=9$, $\theta_{I}=17$, $b_{IE}=1.35$, $\sigma_{IE}=6$, $b_{EI}=1.35$, and $\sigma_{EI}=6$.

### 5.2. Numerical Simulation 2

In [2] (Section 3.3.1), Wilson and Cowan applied their model to the spatial hysteresis in the one-dimensional tissue model. In this experiment, a human subject was exposed to a binocular stimulus. The authors used sharply peaked Gaussian distributions to model the stimuli. The two stimuli were symmetrically moved apart by a small increment and re-summed, and the network response was allowed to reach equilibrium.

Initially, the two peaks (stimuli) were very close; the network response consisted of a single pulse (peak) (see [2] (Section 3.3.1, Figure 13A)). Then, the peaks separated from each other (i.e., the disparity between the two stimuli increased). The network response was a pulse in the middle of the binocular stimulus until a critical disparity was reached. At this stimulus disparity, the single pulse (peak) decayed rapidly to zero, and twin response pulses formed at the locations of the now rather widely separated stimuli; see [2] (Section 3.3.1, Figure 13B).

Following this, the stimuli were gradually moved together again in the same form until they essentially consisted of one peak. However, the network response consisted of two pulses; see [2] (Section 3.3.1, Figure 13C).

The classical Wilson–Cowan model and our *p*-adic version can predict the results of this experiment. We use the function

(21) $${\tilde{h}}_{E}\left(x,t\right)=e^{-{\left(30\left(0.5-m\left(x\right)\right)-0.5t\right)}^{2}}+e^{-{\left(30\left(0.5-m\left(x\right)\right)+0.5t\right)}^{2}}$$

to model the stimuli in the case where the peaks do not move together and

(22) $$h_{E}\left(x,t\right)={\tilde{h}}_{E}\left(x,t\right)1_{\left[0,18\right]}\left(t\right)+{\tilde{h}}_{E}\left(x,36-t\right)1_{\left[18,36\right]}\left(t\right)$$

to model the stimuli in the case where the peaks gradually move together. The function $m:Z_{p}\to R$ is the Monna map; see [38].

**Figure 6:**
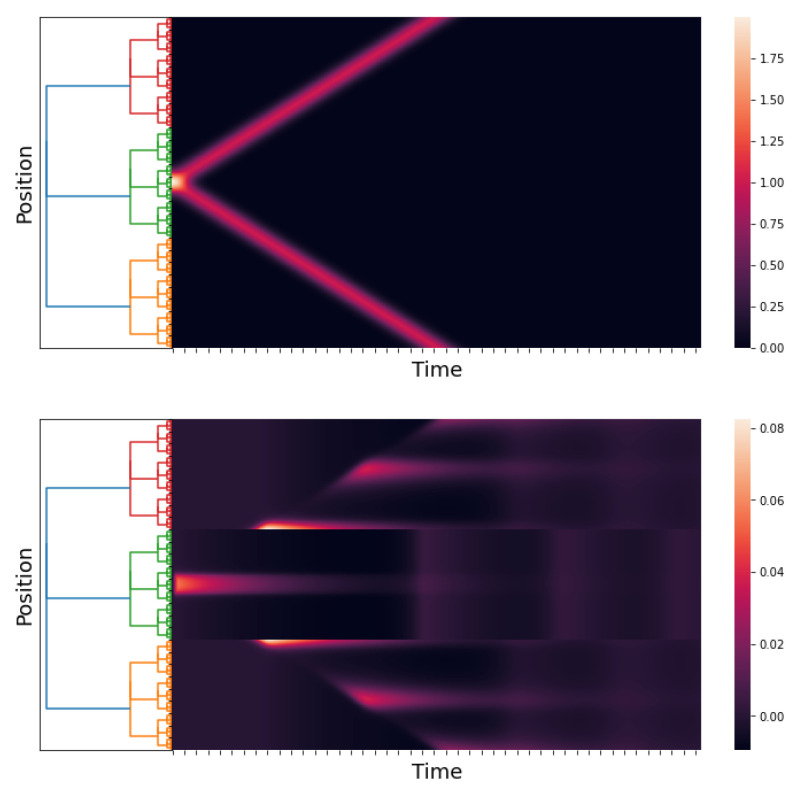
An approximation of ${\tilde{h}}_{E}\left(x,t\right)$ and E(x,t). We take $h_{I}\left(x,t\right)\equiv 0$, p=3, and l=6; the kernels $w_{AB}$ are as in Simulation 1, and $h_{E}\left(x,t\right)$ is as in (21). The time axis goes from 0 to 60 with a step of 0.05. The first figure is the stimuli, and the second figure is the response of the network.

Figure 6 shows the stimuli (see (21)) and the network response when the stimulus peaks are gradually separated. The network response begins with a single pulse. When a critical disparity threshold is reached, the response becomes a twin pulse, which is the prediction of the classical Wilson–Cowan model; see [2] (Section 3.3.1, Figure 13A,B).

**Figure 7:**
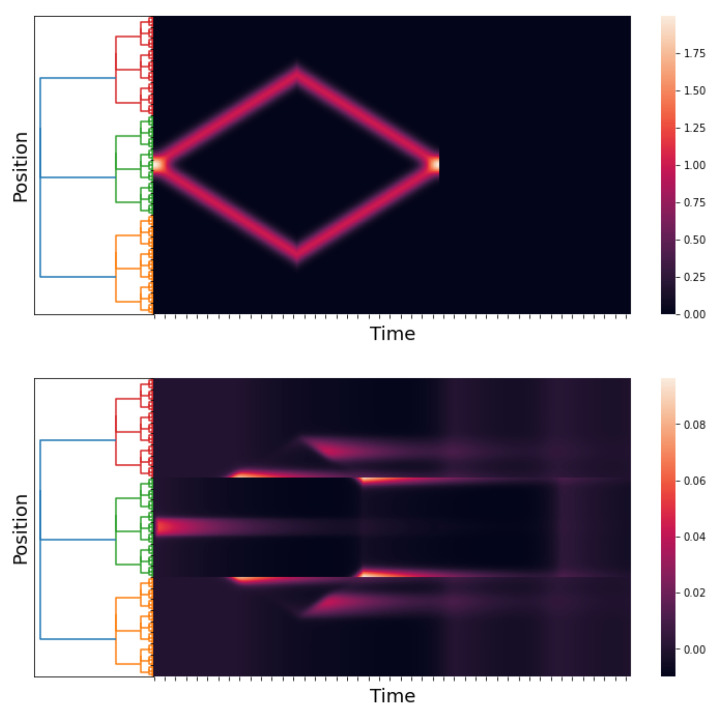
An approximation of $h_{E}\left(x,t\right)$ and E(x,t). We take $h_{I}\left(x,t\right)\equiv 0$, p=3, and l=6; the kernels $w_{AB}$ are as in Simulation 1, and $h_{E}\left(x,t\right)$ is as in (22). The time axis goes from 0 to 60 with a step of 0.05. The first figure is the stimuli, and the second figure is the response of the network.

Figure 7 depicts the stimuli and the network response in the instance where the stimulus peaks gradually split and finally move together. The network response at the end of the experiment consists of twin pulses. This finding is consistent with that of the classical Wilson–Cowan model [2] (Section 3.3.1, Figure 13C).

## 6. p-Adic Kernels and Connection Matrices

There have been significant theoretical and experimental developments in comprehending the wiring diagrams (connection matrices) of the cerebral cortex of invertebrates and mammals over the last thirty years; see, for example, [6,7,8,9,10,11,12,13,14,15,16,17,18,19] and the references therein. The topology of cortical neural networks is described by connection matrices. Building dynamic models from experimental data recorded in connection matrices is a very relevant problem.

**Figure 8:**
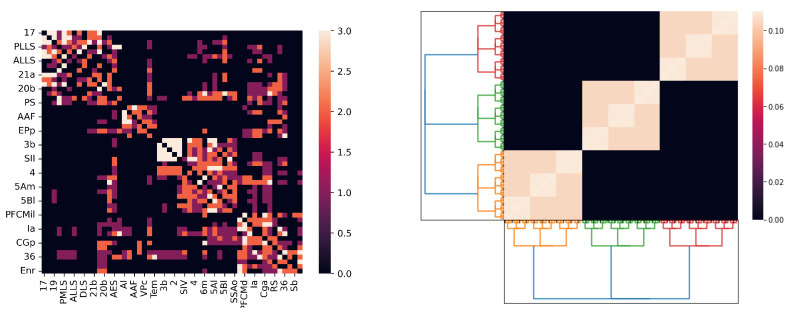
The left matrix is the connection matrix of the cat cortex. The right matrix corresponds to a discretization of the kernel $w_{EE}$ used in Simulation 1.

We argue that our *p*-adic Wilson–Cowan model provides meaningful dynamics on networks whose topology comes from a connection matrix. Figure 8 depicts the connection matrix of the cat cortex (see, e.g., [7,8,9,10,11,12,13,14]) and the matrix of the kernel $w_{EE}$ used in Simulation 1. The *p*-adic methods are relevant only if the connection matrices can be very well approximated for matrices coming from discretizations of *p*-adic kernels. This is an open problem. Here, we show that such an approximation is feasible for the cat cortex connection matrix.

Given an arbitrary matrix *A*, by adding zero entries, we may assume that its size is $p^{k}\times p^{k}$, where *p* is a suitable prime number. We assume that A=${\left[a_{ij}\right]}_{i,j\in G_{k}}$, where $G_{k}$ is the ring of integers modulo $p^{k}$ endowed with the *p*-adic topology, as in the above. This hypothesis means that the connection matrices have an ultrametric nature; this type of matrices appear in connection with complex systems, such as spin glasses; see [23] (Section 4.2) and the references therein. Given an integer *r* satisfying 0≤r≤k, the reduction mod $p^{r}$ map is defined as $i_{0}+i_{1}p+\ldots +i_{k-1}p^{k-1}\to i_{0}+i_{1}p+\ldots +i_{r-1}p^{r-1}$. We now define

$$\begin{array}{ccc} G_{k}\times G_{k} & \underset{\to }{\Pi_{r}} & G_{r}\times G_{r}\\ \left(i,j\right) & \to & \left(i\;\mathrm{mod}p^{r},j\;\mathrm{mod}p^{r}\right). \end{array}$$

**Figure 9:**
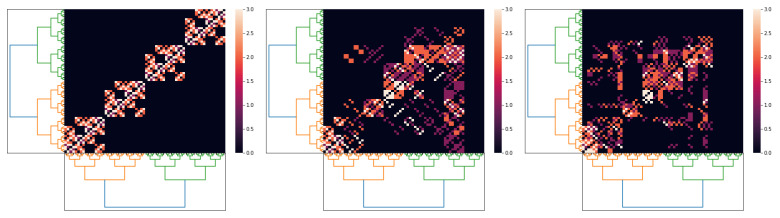
Three *p*-adic approximations for the connection matrix of the cat cortex. We take p=2 and l=6. The first approximation uses r=0; the second, r=3; and the last, r=5.

Map $\Pi_{r}^{-1}$ induces a block decomposition of matrix *A* into $p^{2\left(k-r\right)}$ blocks of size $p^{r}\times p^{r}$. Given $\left(a,b\right)\in G_{r}\times G_{r}$, the corresponding block is $A^{\left(a,b\right)}={\left[a_{ij}\right]}_{\left(i,j\right)\in \Pi_{r}^{-1}\left(a,b\right)}$. Now, we attach to $\left(a,b\right)\in G_{r}\times G_{r}$,

$$\phi_{a,b}\left(x,y\right)=\sum_{l\in G_{r}}\;\sum_{m\in G_{r}}\phi_{a,b}\left(l,m\right)\Omega \left(p^{r}{\left|x-l\right|}_{p}\right)\Omega \left(p^{r}{\left|y-m\right|}_{p}\right)\in D_{r}\left(Z_{p}\times Z_{p}\right),$$

and identify $\phi_{a,b}\left(x,y\right)$ with matrix ${\left[\phi_{a,b}\left(l,m\right)\right]}_{l,m\in G_{r}}$. By using the correspondence

$${\left[\phi_{a,b}\left(l,m\right)\right]}_{l,m\in G_{r}}⟷A^{\left(a,b\right)},$$

we approximate matrix *A* with a kernel $K_{r}\left(x,y\right)$, which is locally translation invariant. More precisely, for each $a,b\in G_{r}$, $K_{r}\left(x,y\right)=\phi_{a,b}\left(x-y\right)$ for all $x\in a+p^{r}Z_{p}$ and $y\in b+p^{r}Z_{p}$. Notice that if r=k, the matrix attached to $K_{r}\left(x,y\right)$ is *A*. See Figure 9. This procedure allows us to incorporate experimental data from connecting matrices into our *p*-adic Wilson–Cowan model.

By using the above procedure, we replace the excitatory–excitatory relation term $w_{EE}*E$ with $\int_{Z_{p}}K_{r}\left(x,y\right)E\left(y\right)dy$ but keep the other kernels as in Simulation 1. For the stimuli, we use $h_{E}=3.5\Omega (p^{2}{\left|x-1\right|}_{p})$, with p=2, l=6, and $h_{I}\left(x\right)=-30$. In Figure 9, we show three different approximations for the cat cortex connection matrix using *p*-adic kernels. The black area in the right matrix in Figure 9 (which corresponds to zero entries) comes from the process of adjusting the size of the origin matrix to $2^{6}\times 2^{6}$.

**Figure 10:**
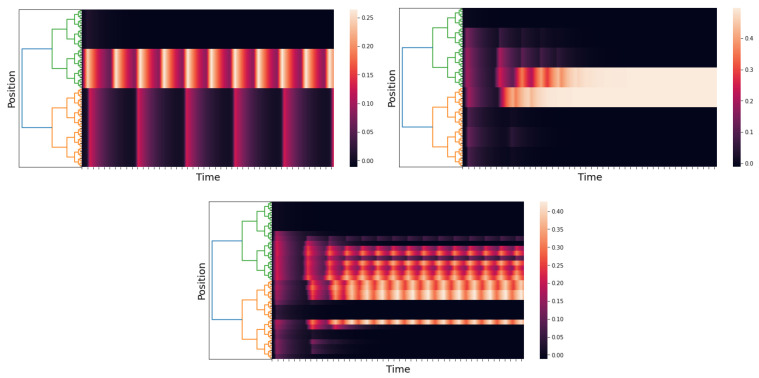
We use p=2 and l=6, and the time axis goes from 0 to 150 with a step of 0.05. The left image uses r=0; the right one uses r=3; and the central one uses r=5.

The corresponding *p*-adic network responses are shown in Figure 10 for different values of *r*. In the case r=0, the interaction among neurons is short range, while in the case r=5, there is long-range interaction. The response in the case r=0 is similar to the one presented in Simulation 1; see Figure 5. When the connection matrix gets close to the cat cortex matrix (see Figure 9), which is when the matrix allows more long-range connections, the response of the network presents more complex patterns (see Figure 10).

## 7. Final Discussion

The Wilson–Cowan model describes interactions between populations of excitatory and inhibitory neurons. This model constitutes a relevant mathematical tool for understanding cortical tissue functionality. On the other hand, in the last twenty-five years, there has been tremendous experimental development in understanding the cerebral cortex’s neuronal wiring in invertebrates and mammalians. Employing different experimental techniques, the wiring patterns can be described by connection matrices. Such a matrix is just an adjacency matrix of a directed graph whose nodes represent neurons, groups of neurons, or portions of the cerebral cortex. The oriented edges represent the strength of the connections between two groups of neurons. This work explores the interplay between the classical Wilson–Cowan model and connection matrices.

Nowadays, it is widely accepted that the networks in the cerebral cortex of mammalians have the small-world property, which means a non-negligible interaction exists between any two groups of neurons in the network. The classical Wilson–Cowan model is not compatible with the small-world property. We show that the original Wilson–Cowan model can be formulated on any topological group, and the Cauchy problem for the underlying equations of the model is well posed. We give an argument showing that the small-world property requires that the group be compact, and consequently, the classical model should be discarded. In practical terms, the classical Wilson–Cowan model cannot incorporate the experimental information contained in connection matrices. We propose a *p*-adic Wilson–Cowan model, where the neurons are organized in an infinite rooted tree. We present numerical experiments showing that this model can explain several phenomena, similarly to the classical model. The new model can incorporate experimental information coming from connection matrices.

## References

[1] Wilson H.R., Cowan J.D. (1972). Excitatory and inhibitory interactions in localized populations of model neurons. Biophys. J..

[2] Wilson H.R., Cowan J.D.A. (1973). Mathematical theory of the functional dynamics of cortical and thalamic nervous tissue. Kybernetik.

[3] Stephen C., Peter B.G., Roland P., James W. (2014). Neural Fields: Theory and Applications.

[4] Thierry C., Alain H. (1998). An Introduction to Semilinear Evolution Equations.

[5] Milan M. (1998). Applied Functional Analysis and Partial Differential Equations.

[6] Sporns O., Tononi G., Edelman G.M. (2000). Theoretical neuroanatomy: Relating anatomical and functional connectivity in graphs and cortical connection matrices. Cereb. Cortex.

[7] Scannell J.W., Burns G.A., Hilgetag C.C., O’Neil M.A., Young M.P. (1999). The connectional organization of the cortico-thalamic system of the cat. Cereb. Cortex.

[8] Sporns O. (2006). Small-world connectivity, motif composition, and complexity of fractal neuronal connections. Biosystems.

[9] Sporns O., Honey C.J. (2006). Small worlds inside big brains. Proc. Natl. Acad. Sci. USA.

[10] Hilgetag C.C., Goulas A. (2016). Is the brain really a small-world network?. Brain Struct. Funct..

[11] Muldoon S.F., Bridgeford E.W., Bassett D.S. (2016). Small-World Propensity and Weighted Brain Networks. Sci. Rep..

[12] Bassett D.S., Bullmore E.T. (2017). Small-World Brain Networks Revisited. Neuroscientist.

[13] Akiki T.J., Abdallah C.G. (2019). Determining the Hierarchical Architecture of the Human Brain Using Subject-Level Clustering of Functional Networks. Sci. Rep..

[14] Scannell J.W., Blakemore C., Young M.P. (1995). Analysis of connectivity in the cat cerebral cortex. J. Neurosci..

[15] Fornito A., Zalesky A., Bullmore E. (2016). Connectivity Matrices and Brain Graphs. Fundamentals of Brain Network Analysis.

[16] Sporns O. (2016). Networks of the Brain.

[17] Swanson L.W., Hahn J.D., Sporns O. (2017). Organizing principles for the cerebral cortex network of commissural and association connections. Proc. Natl. Acad. Sci. USA.

[18] Demirtaş M., Burt J.B., Helmer M., Ji J.L., Adkinson B.D., Glasser M.F., Van Essen D.C., Sotiropoulos S.N., Anticevic A., Murray J.D. (2019). Hierarchical Heterogeneity across Human Cortex Shapes Large-Scale Neural Dynamics. Neuron.

[19] Škoch A., Rehák Bučkovxax B., Mareš J., Tintěra J., Sanda P., Jajcay L., Horxaxxcxek J., Španiel F., Hlinka J. (2022). Human brain structural connectivity matrices-ready for modeling. Sci. Data.

[20] Avetisov V.A., Bikulov A.K., Osipov V.A. (2003). p-Adic description of characteristic relaxation in complex systems. J. Phys. A.

[21] Avetisov V.A., Bikulov A.H., Kozyrev S.V., Osipov V.A. (2002). p-Adic models of ultrametric diffusion constrained by hierarchical energy landscapes. J. Phys. A.

[22] Parisi G., Sourlas N. (2000). p-Adic numbers and replica symmetry breaking. Eur. Phys. J. B.

[23] Khrennikov A., Kozyrev S., Zúñiga-Galindo W.A. (2018). Ultrametric Equations and Its Applications: Encyclopedia of Mathematics and Its Applications 168.

[24] Zúñiga-Galindo W.A. (2022). Eigen’s paradox and the quasispecies model in a non-Archimedean framework. Phys. A Stat. Mech. Its Appl..

[25] Zúñiga-Galindo W.A. (2022). Ultrametric diffusion, rugged energy landscapes, and transition networks. Phys. A Stat. Mech. Its Appl..

[26] Zúñiga-Galindo W.A. (2020). Reaction-diffusion equations on complex networks and Turing patterns, via p-adic analysis. J. Math. Anal. Appl..

[27] Zambrano-Luna B.A., Zuniga-Galindo W.A. (2023). *p*-Adic cellular neural networks. J. Nonlinear Math. Phys..

[28] Zambrano-Luna B.A., Zúñiga-Galindo W.A. (2023). *p*-Adic cellular neural networks: Applications to image processing. Phys. D Nonlinear Phenom..

[29] Vladimirov V.S., Volovich I.V., Zelenov E.I. (1994). p-Adic Analysis and Mathematical Physics.

[30] Albeverio S., Khrennikov A., Shelkovich V.M. (2010). Theory ofp-Adicdistributions: Linear and Nonlinear Models.

[31] Kochubei A.N. (2001). Pseudo-Differential Equations and Stochastics over Non-Archimedean Fields.

[32] Taibleson M.H. (1975). Fourier Analysis on Local Fields.

[33] Bocardo-Gaspar M., García-Compeán H., Zúñiga-Galindo W.A. (2019). Regularization of p-adic string amplitudes, and multivariate local zeta functions. Lett. Math. Phys..

[34] Koblitz N. (1984). p-Adic Numbers, p-Adic Analysis, and Zeta-Functions. Graduate Texts in Mathematics No. 58.

[35] Chistyakov D.V. (1996). Fractal geometry of images of continuous embeddings of p-adic numbers and solenoids into Euclidean spaces. Theor. Math. Phys..

[36] Halmos P. (1950). Measure Theory.

[37] Zúñiga-Galindo W.A. (2018). Non-Archimedean Reaction-Ultradiffusion Equations and Complex Hierarchic Systems. Nonlinearity.

[38] Monna A.F. (1952). Sur une transformation simple des nombres p-adiques en nombres réels. Indag. Math..

